# Supplementary material for: Influence of phragmites density, algal concentration and water velocity on cyanobacterial bloom dynamics
Source: PeerJ. 2025 Jul 16;13:e19704. doi: 10.7717/peerj.19704 (PMC12275901; doi:10.7717/peerj.19704)
Supplement: Supplemental Information 1 — Our orthogonal study conducted 6 times over a one-month period, two repetitions were taken for each sample to acquire the average. Sal, DO, TN, TP, OM and NH3-N were chosen to represent water quality changes during cyanobacterial decomposition. S represents the overall standard deviation of each group of samples. It indicates the degree of influence that different levels of factors have on water quality indicators. [file peerj-13-19704-s001.pdf]

Table S1. Time distribution of water quality change under different conditions. Our orthogonal study conducted 6 times over a one-month period, two repetitions were taken for each sample to acquire the average. Sal, DO, TN, TP, OM and NH_3-N were chosen to represent water quality changes during cyanobacterial decomposition. S represents the overall standard deviation of each group of samples. It indicates the degree of influence that different levels of factors have on water quality indicators.

	group	D0	D1	D4	D10	D20	D30	S
DO (mg/L)	1	4.865	6.21	7.245	12.165	10.83	11.3	2.78
	2	2.725	13.35	7.65	10.705	10.725	10.035	3.34
	3	0.32	15.745	8.11	12.645	12.42	10.485	4.89
	4	2.855	10.75	6.365	10.74	11.67	10.72	3.18
	5	2.285	3.66	4.695	12.71	10.965	10.66	4.06
	6	0.42	13.93	6.65	17.655	12.49	11.555	5.54
	7	3.09	10.255	6.88	13.965	12.245	12.05	3.70
	8	2.525	14.235	8.48	8.715	10.565	10.17	3.50
	9	0.495	1.205	2.015	15.59	10.375	17.37	6.94
Sal	1	0.20	0.21	0.22	0.28	0.23	0.24	0.03
	2	0.20	0.20	0.21	0.29	0.26	0.31	0.04
	3	0.22	0.21	0.22	0.28	0.22	0.24	0.02
	4	0.21	0.22	0.28	0.32	0.22	0.26	0.04
	5	0.21	0.21	0.23	0.29	0.24	0.25	0.03
	6	0.22	0.20	0.21	0.25	0.21	0.21	0.02
	7	0.21	0.23	0.26	0.30	0.23	0.25	0.03
	8	0.21	0.20	0.23	0.29	0.26	0.30	0.04
	9	0.22	0.23	0.25	0.27	0.22	0.20	0.02
TN (mg/L)	1	8.04	5.34	4.82	3.19	2.08	1.53	2.20
	2	12.60	8.67	6.26	5.43	2.21	1.46	3.79
	3	34.20	29.20	20.63	14.15	14.43	6.95	9.33
	4	10.86	5.68	4.34	3.77	2.57	1.32	3.05
	5	12.38	10.19	12.06	7.32	3.35	2.48	3.93
	6	21.93	22.00	20.72	20.17	16.25	8.38	4.81
	7	6.35	4.33	3.53	3.14	2.18	1.11	1.65
	8	10.83	8.92	7.34	4.42	2.97	2.32	3.13
	9	36.09	22.67	18.34	41.73	24.48	12.51	10.03
TP (mg/L)	1	0.823	0.578	0.524	0.288	0.132	0.080	0.26
	2	1.349	1.009	0.747	0.586	0.235	0.127	0.42
	3	3.342	2.964	2.107	1.248	1.348	0.528	0.99
	4	1.077	0.611	0.440	0.309	0.234	0.075	0.32
	5	1.321	1.129	1.327	0.812	0.278	0.210	0.46
	6	2.398	2.368	2.386	2.192	1.626	0.780	0.59
	7	0.692	0.542	0.408	0.333	0.218	0.046	0.21
	8	1.081	0.952	0.780	0.407	0.247	0.199	0.34
	9	3.737	2.611	2.452	4.600	2.711	1.288	1.04

OM (mg/L)	1	1117.50	1167.29	752.55	241.76	129.75	65.25	455.65
	2	1761.25	1700.00	1215.40	728.00	191.50	112.00	660.54
	3	4021.00	3087.50	3514.17	2194.00	1908.00	823.75	1071.67
	4	1420.00	1134.11	770.00	384.29	166.88	79.82	495.02
	5	1688.21	2145.00	1783.56	965.09	307.81	270.38	728.82
	6	3033.75	2405.00	3763.59	3261.67	2163.33	1198.00	833.24
	7	912.75	1145.71	576.67	326.94	140.50	40.77	399.97
	8	1503.33	1608.33	1444.25	724.75	319.05	267.70	561.78
	9	4352.50	2676.67	3184.44	4883.33	3122.50	1744.17	1037.97
NH_3-N (mg/L)	1	0.25	0.18	0.34	0.38	0.29	0.30	0.06
	2	0.28	0.19	0.15	0.34	0.23	0.20	0.06
	3	0.41	0.37	0.29	0.58	0.46	1.14	0.28
	4	0.31	0.25	0.25	0.39	0.33	0.27	0.05
	5	0.32	0.28	0.38	0.48	0.42	0.36	0.06
	6	0.41	0.37	0.32	0.40	0.58	1.15	0.29
	7	0.38	0.19	0.20	0.33	0.27	0.27	0.07
	8	0.24	0.27	0.24	0.43	0.33	0.32	0.07
	9	0.42	0.30	0.50	0.84	0.98	1.70	0.47
Chl-α (μg/L)	1	678.65	333.74	202.28	105.57	29.38	6.89	230.30
	2	1362.06	668.87	325.73	182.44	67.53	20.66	464.85
	3	3151.12	2430.41	1786.44	714.54	619.95	134.39	1074.38
	4	1241.57	374.08	171.17	118.73	46.86	9.19	425.40
	5	1331.10	816.62	626.46	326.41	92.24	42.76	447.81
	6	2546.14	1971.30	1646.92	1020.41	609.45	189.13	806.98
	7	468.87	234.17	149.40	114.09	43.13	3.12	153.26
	8	999.84	653.32	379.38	114.42	74.02	43.69	350.27
	9	2894.06	2082.12	1711.67	2925.47	1446.95	507.04	841.56
