# Supplementary material for: Influence of phragmites density, algal concentration and water velocity on cyanobacterial bloom dynamics
Source: PeerJ. 2025 Jul 16;13:e19704. doi: 10.7717/peerj.19704 (PMC12275901; doi:10.7717/peerj.19704)
Supplement: Supplemental Information 2 — Diversity indexes, including Richness, Chao1, ACE, Shannon, Simpson and Invsimpson, were calculated using a normalized sequencing depth of 6547 reads. Two repetitions were taken for each sample to acquire the average. S represents the overall standard deviation of each group of samples. [file peerj-13-19704-s002.pdf]

Table S2. The Alpha-diversity index of each sample. Diversity indexes, including Richness, Chao1, ACE, Shannon, Simpson and Invsimpson, were calculated using a normalized sequencing depth of 6547 reads. Two repetitions were taken for each sample to acquire the average. S represents the overall standard deviation of each group of samples.

	group	D0	D1	D4	D10	D20	D30	S
Richness	1	973	918	997	919	872	793	66.86
	2	954	835	922	777	851	818	60.65
	3	890	944	832	772	790	691	81.92
	4	986	963	993	916	1011	851	54.67
	5	936	971	960	796	876	744	85.03
	6	976	931	870	740	895	680	104.79
	7	1008	954	1033	895	984	656	126.66
	8	1054	917	900	814	751	841	95.47
	9	957	1047	864	884	770	842	88.16
Chao1	1	1277	1270	1279	1252	1176	1090	69.64
	2	1248	1131	1180	1043	1090	1093	66.99
	3	1199	1365	1103	1029	1022	969	133.48
	4	1323	1344	1239	1260	1351	1203	55.91
	5	1245	1263	1262	1101	1169	1077	75.88
	6	1301	1262	1176	1056	1159	921	127.19
	7	1428	1277	1299	1209	1284	1019	123.36
	8	1409	1314	1210	1080	1059	1039	139.01
	9	1237	1358	1096	1146	1037	1152	103.22
ACE	1	1307	1299	1310	1282	1218	1149	58.86
	2	1266	1177	1202	1065	1168	1114	63.77
	3	1239	1358	1141	1078	1047	975	127.30
	4	1352	1382	1299	1306	1433	1207	71.14
	5	1313	1332	1284	1100	1202	1071	101.91
	6	1342	1281	1176	1051	1203	982	124.15
	7	1446	1322	1355	1244	1339	1026	131.78
	8	1455	1331	1225	1126	1069	1091	139.06
	9	1300	1421	1119	1182	1064	1179	118.24
Invsimpsom	1	8	19	45	37	15	25	12.61
	2	10	17	18	17	13	22	3.83
	3	17	29	16	19	15	4	7.23
	4	7	9	17	15	16	13	3.38
	5	8.61	35.19	28.93	22.62	11.74	5.43	10.94
	6	19.76	30.60	19.77	11.19	15.94	3.79	8.26
	7	7.49	9.50	27.20	8.09	25.21	11.28	8.18
	8	27.86	22.83	17.32	8.48	5.50	8.80	8.20
	9	24.21	24.27	24.15	23.34	6.12	13.10	7.08
