# Supplementary material for: Influence of phragmites density, algal concentration and water velocity on cyanobacterial bloom dynamics
Source: PeerJ. 2025 Jul 16;13:e19704. doi: 10.7717/peerj.19704 (PMC12275901; doi:10.7717/peerj.19704)
Supplement: Supplemental Information 3 — K value is the sum of the results of three levels of factors and reflects the influence of different levels and that R value the influence of different factors. S represents the overall standard deviation of each group of samples. It indicates the degree of influence that different levels of factors have on water quality indicators. [file peerj-13-19704-s003.pdf]

Table S3. The effects of three factors on water quality and bacterial diversity during cyanobacteria degradation were examined by orthogonal analysis. K value is the sum of the results of three levels of factors and reflects the influence of different levels and that R value the influence of different factors. S represents the overall standard deviation of each group of samples. It indicates the degree of influence that different levels of factors have on water quality indicators.

a)

	Phyragmites density	Algal concentration	Water velocity	Index S_{TN}
1	low	low	low	2.20
2	low	medium	medium	3.79
3	low	high	high	9.33
4	medium	low	medium	3.05
5	medium	medium	low	3.93
6	medium	high	high	4.81
7	high	low	high	1.65
8	high	medium	medium	3.13
9	high	high	low	10.03
K_1	**15.32**	6.9	**16.16**	
K_2	11.79	10.85	9.97	
K_3	14.81	**24.17**	15.79	$P_1A_3W_1$
$k_1=K_1/3$	5.11	2.3	5.39	
$k_2=K_2/3$	3.93	3.62	3.33	
$k_3=K_3/3$	4.94	8.06	5.27	
R	1.18	**5.76**	2.06	A>W>P

b)

	Phyragmites density	Algal concentration	Water velocity	Index S_{TP}
1	low	low	low	0.26
2	low	medium	medium	0.42
**3**	**low**	**high**	**high**	**0.99**
4	medium	low	medium	0.32
5	medium	medium	low	0.46
6	medium	high	high	0.59
7	high	low	high	0.21
8	high	medium	medium	0.34
9	high	high	low	1.04
K_1	**1.67**	0.79	1.76	
K_2	1.37	1.22	1.08	
K_3	1.59	**2.62**	**1.79**	$P_1A_3W_3$
$k_1=K_1/3$	0.56	0.26	0.59	
$k_2=K_2/3$	0.46	0.41	0.36	

k_3=K_3/3	0.53	0.87	0.6	
R	0.1	**0.61**	0.24	A>W>P

c)

	Phyragmites density	Algal concentration	Water velocity	Index
				**S_{OM}**
1	low	low	low	455.65
2	low	medium	medium	660.54
**3**	**low**	**high**	**high**	**1071.67**
4	medium	low	medium	495.02
5	medium	medium	low	728.82
6	medium	high	high	833.24
7	high	low	high	399.97
8	high	medium	medium	561.78
9	high	high	low	1037.97
K_1	**2187.86**	1350.64	2222.44	$P_1A_3W_3$
K_2	2057.08	1951.14	1717.34	
K_3	1999.72	**2942.88**	**2304.88**	
k_1=K_1/3	729.29	450.21	740.81	
k_2=K_2/3	685.69	650.38	572.45	
k_3=K_3/3	666.57	980.96	768.29	
R	62.72	**530.75**	195.84	A>W>P

d)

	Phyragmites density	Algal concentration	Water velocity	Index
				**$S_{NH3\text{-}N}$**
1	low	low	low	0.06
2	low	medium	medium	0.06
3	low	high	high	0.28
4	medium	low	medium	0.05
5	medium	medium	low	0.06
6	medium	high	high	0.29
7	high	low	high	0.07
8	high	medium	medium	0.07
9	high	high	low	0.47
K_1	0.4	0.18	0.59	$P_3A_3W_3$
K_2	0.4	0.19	0.18	
K_3	**0.61**	**1.04**	**0.64**	
k_1=K_1/3	0.13	0.06	0.2	
k_2=K_2/3	0.13	0.06	0.06	
k_3=K_3/3	0.2	0.35	0.21	
R	0.07	**0.29**	0.15	A>W>P

e）

	Phyragmites density	Algal concentration	Water velocity	Index S_{Chao1}
1	low	low	low	69.64
2	low	medium	medium	66.99
3	low	high	high	133.48
4	medium	low	medium	55.91
5	medium	medium	low	75.88
6	medium	high	high	127.19
7	high	low	high	123.36
8	high	medium	medium	139.01
9	high	high	low	103.22
K_1	270.11	248.91	248.74	$P_3A_3W_3$
K_2	258.98	281.88	261.91	
K_3	**365.59**	**363.89**	**384.03**	
$k_1=K_1/3$	90.04	82.97	82.91	
$k_2=K_2/3$	86.33	93.96	87.3	
$k_3=K_3/3$	121.86	121.3	128.01	
R	35.53	38.33	**45.04**	W>A>P

f）

	Phyragmites density	Algal concentration	Water velocity	Index S_{β}
1	low	low	low	0.169
2	low	medium	medium	0.159
3	low	high	high	0.182
4	medium	low	medium	0.211
5	medium	medium	low	0.162
6	medium	high	high	0.175
7	high	low	high	0.205
8	high	medium	medium	0.18
9	high	high	low	0.145
K_1	0.51	**0.585**	0.467	$P_2A_1W_3$
K_2	**0.548**	0.501	0.55	
K_3	0.53	0.502	**0.562**	
$k_1=K_1/3$	0.17	0.195	0.159	
$k_2=K_2/3$	0.182	0.167	0.183	
$k_3=K_3/3$	0.177	0.167	0.187	
R	0.012	**0.028**	0.026	A>W>P

g）

	Phyragmites density	Algal concentration	Water velocity	Index S_{BCC}

**1**	**low**	**low**	**low**	**11.08%**
2	low	medium	medium	8.4%
3	low	high	high	5.7%
4	medium	low	medium	7.05%
5	medium	medium	low	7.39%
6	medium	high	high	7.3%
7	high	low	high	9.09%
8	high	medium	medium	6.93%
9	high	high	low	6.93%
K_1	**25.18%**	**27.22%**	**25.4%**	
K_2	21.74%	22.72%	22.38%	
K_3	22.95%	19.93%	22.09%	
$k_1=K_1/3$	8.39%	9.07%	8.47%	$\mathrm{P_1A_1W_1}$
$k_2=K_2/3$	7.25%	7.57%	7.46%	
$k_3=K_3/3$	7.65%	6.64%	7.36%	
$\boldsymbol{R}$	1.14%	**2.43%**	1.11%	A>P>W
